# Variations in plasmid transfer in *Acinetobacter baumannii*: insights from epigenetics, strain properties, and experimental conditions

**DOI:** 10.1128/spectrum.03487-25

**Published:** 2026-04-27

**Authors:** Jonathan Koong, Laurence D. W. Luu, Iain G. Duggin, Mehrad Hamidian

**Affiliations:** 1 Australian Institute for Microbiology & Infection, University of Technology Sydney 1994, Ultimo, New South Wales, Australia; 2 School of Biotechnology and Biomolecular Sciences, University of New South Wales 98492, Sydney, New South Wales, Australia; 3 School of Life Sciences, University of Technology Sydney 541010, Sydney, New South Wales, Australia; Université de Lorraine, Nancy, France

**Keywords:** *Acinetobacter baumannii*, plasmid, conjugation, transformation, conjugation, methylation and antimicrobial resistance

## Abstract

Plasmid-mediated horizontal gene transfer is a driver of antibiotic resistance dissemination in *Acinetobacter baumannii*. Here, we investigated the extent to which intrinsic host factors influence plasmid uptake across diverse *A. baumannii* isolates using electro-transformation and conjugative transfer assays. To enable comparisons, electro-transformation conditions were optimized to minimize technical variability and reveal strain-dependent differences in plasmid uptake. Under the conditions used here, substantial variation in transfer efficiency was observed, including between closely related strains, indicating that plasmid acquisition is strongly influenced by the recipient genetic background. Comparative analyses of electro-transformation and conjugation demonstrated that while experimental parameters such as competent cell density, DNA input, and recipient-to-donor ratios affect transfer efficiency, these factors do not overcome inherent strain-specific barriers. Genome and methylome analyses also revealed extensive diversity in restriction-modification systems and DNA methylation patterns among recipient strains. Moreover, plasmids exhibited altered methylation profiles following transfer into new hosts, consistent with host-driven epigenetic modification. These findings indicate that plasmid transfer efficiency in *A. baumannii* is determined primarily by strain-specific genetic and epigenetic features rather than transferable experimental conditions. This variability has important implications for interpreting plasmid mobility, host range, and resistance dissemination within this clinically significant species.

**IMPORTANCE:**

Plasmid-mediated gene transfer is one of the major drivers of antibiotic resistance in *Acinetobacter baumannii*; however, plasmid transfer protocols remain inconsistent and strain-dependent. By assessing electroporation and conjugation across diverse strains, we identify key experimental and genomic factors, such as test and strain differences, including restriction-modification systems and epigenetic signatures, that influence plasmid uptake. These findings offer practical guidance for optimizing plasmid transfer protocols and highlight strain-level barriers that impact resistance gene dissemination in this critical microorganism.

## INTRODUCTION

*Acinetobacter baumannii* is an opportunistic bacterial pathogen and a significant global public health threat due to extensive antimicrobial resistance (AMR) causing treatment failures. The widespread dissemination of AMR in *A. baumannii* is primarily due to gene acquisition via mobile genetic elements, including plasmids (extra-chromosomal DNA molecules) (1–4). *A. baumannii* plasmids are increasingly being recognized for their significant role in spreading numerous antibiotic resistance genes (ARGs) (5–7). Thus, understanding the transferability of plasmids is essential for evaluating their capacity to disseminate genetic material, including antibiotic resistance and virulence genes (8). Large conjugative plasmids that encode a complete set of transfer functions often move via conjugative transfer, which is assessed in the laboratory via the classical mating assay (also known as the conjugation or matting assay) (9). However, smaller plasmids, which lack a complete set of *tra* genes, often move by co-transfer alongside a conjugative plasmid or via transformation, which are assessed via mobilization (using matting assays) or transformation assays, respectively (8).

Previously, we demonstrated the transferability of multiple *A. baumannii* conjugative plasmids, predominantly RP-T1 types (formerly Aci6, RepPriCT_1; Pfam03090) (10–13), as well as those related to pAB3 and pA297-3 (14, 15), using classic mating assays with protocols primarily adapted from Enterobacterales (8). However, conjugative transfer conditions remain to be optimized for *A. baumannii* plasmids. Like conjugation, transformation (either via natural transformation conditions or electro-transformation) is also influenced by multiple factors, including DNA quantity, temperature, cell density, solution composition, and bacterial growth phase (16, 17). In 2016, Yildirim and colleagues assessed various parameters for transformation with an engineered shuttle vector (pWH1266) between *Escherichia coli* and *Acinetobacter* species (18). They found that the highest efficiency occurred during the stationary growth phase of the bacterium (OD_600_ 6.0) using 25 ng of plasmid DNA (18). However, these transformation conditions might vary for different plasmids with different accessory genes and therefore influence their transfer potential. In addition, a recent study has shown that the original host of both genomic and plasmid DNA can introduce variability in the efficiency of transformation in *A. baumannii* (19). They concluded that the restriction-modification (RM) systems found in both the host and recipient cells limited transformability (19). When the modification enzyme was removed, plasmid acquisition efficiency was increased, suggesting that the epigenetic modifications of incoming DNA are an important factor for DNA uptake in *A. baumannii* (19, 20).

In this study, we examined the impacts of various host, plasmid, and experimental variables, including the use of different recipient strains, on the transformation efficiency (both electroporation and natural methods) and conjugation of multiple *A. baumannii* plasmids, which play a significant role in the spread of antibiotic resistance genes among major globally distributed clones (such as ST1 and ST2). Furthermore, to enhance our understanding of plasmid host range, we assessed the transfer efficiency of two prevalent *A. baumannii* plasmids both within the *Acinetobacter* genus and across other genera. The findings presented here offer new insights for future investigations into plasmid-mediated AMR transmission and plasmid transfer mechanisms in *Acinetobacter*.

## MATERIALS AND METHODS

### Bacterial strains

Unless otherwise specified, all cultures in liquid or solid media were grown in lysogeny broth (LB), containing 10 g/L NaCl. A set of seven diverse *A. baumannii* isolates was used as plasmid donors and recipients for experimental work in this study (Table 1). Donor strains (*n* = 2) were multidrug-resistant strains carrying plasmids of interest, while recipient strains (*n* = 5) were largely antibiotic-susceptible strains with distinct intrinsic antibiotic selective markers (Table 1). In addition, four non-*baumannii Acinetobacter* strainsand four non-*Acinetobacter* gram-negative strains, including *E. coli* K12*, Pseudomonas aeruginosa* PAO1*, Stenotrophomonas maltophilia* CF13, and *Morganella morganii* 208.01, were also used to test the plasmid host range (Table 2).

**TABLE 1:** *A. baumannii* strains used in this study^*d*^

Strain	Country	Year	Source	Chromosome/Plasmids	Size (bp)	Antibiotic resistance genes	Antibiotic resistance phenotype^*a*^	Role	GenBank Accession no.	Reference
ACICU	Italy	2005	Blood	Chromosome	3,919,274	*oxa20, aacA4, sul1*	STR, SPT, CTX, CAZ, AMK, TOB, NET, SUL	Donor	CP031380.1	(21)
				pACICU1b	24,268	*oxa58*	IPM, MEM		CP031381.2	
				pACICU2	70,101	*aphA6*	AMK, KAN		CP031382.1	
A297	Netherlands	1984	nk^*b*^	Chromosome	4,109,411	*tetA, catA1, bla*_TEM_ *, sul1, aphA1, dfrA1*	STR, SPT, MEM, TET, CHL, CAZ, CTX, SUL, KAN, NEO, TMP	Donor	CP178354.1	(22)
				pA297-1 (pRAY*)	6,078	*aadB*	TOB, GEN, KAN		CP178355.1	
				pA297-2	8,731	–			CP178356.1	
				pA297-3	200,633	*sul2, strAB*	SUL, STR, SPT		CP178357.1	
G7	Australia	2003	nk	Chromosome	4,087,022	*tetA, catA1, bla*_TEM_ *, sul1, aphA1, aacC, aadA*	STR, SPT, MEM, TET, CHL, CAZ, CTX, SUL, KAN, NEO, GEN	Donor	CP175642.1	(12)
				pAB-G7-1	8,731	–			CP175644.1	
				pAB-G7-2	70,100	*aphA6*	AMK, KAN		CP175643.1	
D36	Australia	2008	Wound	Chromosome	4,063,596	*oxa23*	IPM, MEM, CAZ, CTX	Donor	CP012952.1	(23)
				pD36-1	4,754	–			CP012953.1	
				pD36-2 (pRAY*)	6,078	*aadB*	TOB, GEN, KAN		CP012954.1	
				pD36-3	9,276	–			CP012955.1	
				pD36-4	47,457	*sul2*, *aphA1*	KAN, NEO, SUL		CP012956.1	
AB307-0294^RifR^	U.S.	1994	Blood	Chromosome	3,759,495	–	STR, SPT RIF	Recipient	CP001172.2	(21)
ATCC17978^SuS, RifR^		1951	Meningitis	Chromosome	3,857,743	–	STR, SPT, RIF	Recipient	CP012004.1	(24)
				pAB1	13,408	–	–		CP000522.1	
				pAB2	11,302	–	–		CP000523.1	
				pAB3^*c*^	148,955	–			CP012005.1	
Ax270	Lebanon	2012	Cat	Chromosome	3,764,882	–	STR, SPT, NAL	Recipient	CP049240.1	(25)
Ex003^RifR^	Lebanon	2012	Water	Chromosome	3,935,232	–	STR, SPT, RIF	Recipient	CP049314.1	(25)
				pEx003	11,844	–	–		CP049315.1	
SAAb472^RifR^	Australia	2019	Lake	Chromosome	3,809,697	–	STR, SPT, RIF	Recipient	CP127906.1	(26)

^*a*^ Antibiotic acronyms: streptomycin (STR), spectinomycin (SPT), cefotaxime (CTX), ceftazidime (CAZ), amikacin (AMK), tobramycin (TOB), netilmicin (NET), sulfamethoxazole (SUL), imipenem (IPM), meropenem (MEM), kanamycin (KAN), tetracycline (TET), chloramphenicol (CHL), neomycin (NEO), trimethoprim (TMP), rifampicin (RIF), and nalidixic acid (NAL).

^*b*^ not recorded.

^*c*^ pAB3 (containing *sul2*) cured in this study and used as a sulfonamide-susceptible recipient strain.

^*d*^ "–" indicates "no ARG".

**TABLE 2:** Non-*A*. *baumannii* strains used in this study^*b*^

Species	Strain	Country	Year	Source	Antibiotic resistance	Accession number	Reference
*Acinetobacter gerneri* ^RifR^	SAAg309	Australia	2019	IW^*a*^	AMP, SAM, SPT, STR, TOB, GEN, SUL, RIF, NAL, FFC, TET	JASVDU000000000	(26)
*Acinetobacter towneri*	SAAt401	Australia	2019	IW	TMP, NAL, TET	CP127892	(26)
*Acinetobacter johnsonii*	SAAj643	Australia	2019	IW	CAZ, SPT, TMP, NAL	JASVDX000000000	(26)
*Acinetobacter chinensis*	SAAc573	Australia	2019	IW	AMP, CTX, MEM, CAZ, SAM, SPT, STR, TMP, CRO, TET	CP127923	(26)
*Escherichia coli*	K12 (DH5α)	USA	<1993	Lab strain	NAL	CP076470.1	(27)
*Pseudomonas aeruginosa*	PAO1	Australia	1954	Wound	AMP, CTX, SAM, SPT, KAN, RIF, TMP, NAL, FFC, CHL	AE004091.2	(28)
*Stenotrophomonas maltophilia*	CF13	Australia	2011	Sputum	IPM, AMP, CTX, MEM, CAZ, SAM, TOB, SPT, KAN, TMP, CRO, NAL, CIP, FFC, CHL	CP052863.1	(29)
*Morganella morganii*	208.01^RifR^	Australia	2019	Bird	RIF	This study	This study

^*a*^ Influent wastewater.

^*b*^ IPM, imipenem; MEM, meropenem; AMP, ampicillin; CTX, cefotaxime; CAZ, ceftazidime; SAM, ampicillin/sulbactam; SPT, spectinomycin; STR, streptomycin; SUL, sulfamethoxazole; RIF, rifampicin; TMP, trimethoprim; FFC, florfenicol; CHL, chloramphenicol; TET, tetracycline; NAL, nalidixic acid; GEN, gentamicin; and CIP, ciprofloxacin.

### Antimicrobial resistance tests

Antibiotic resistance profiles of the bacterial strains were determined using the Calibrated Dichotomous Sensitivity test (CDS) (30), with the following antibiotic discs obtained from Oxoid, New Hampshire, UK: imipenem (10 µg), ampicillin (25 µg), cefotaxime (30 µg), meropenem (10 µg), ceftazidime (30 µg), ampicillin/sulbactam (20 µg), tobramycin (10 µg), spectinomycin (25 µg), gentamicin (10 µg), netilmicin (30 µg), neomycin (30 µg), kanamycin (30 µg), streptomycin (25 µg), amikacin (30 µg), sulfamethoxazole (100 µg), rifampicin (30 µg), trimethoprim (5 µg), ceftriaxone (30 µg), nalidixic acid (30 µg), ciprofloxacin (5 µg), florfenicol (30 µg), chloramphenicol (30 µg), and tetracycline (30 µg) (Table S1). Briefly, strains of interest, including *Acinetobacter* and non-*Acinetobacter* strains, were streaked onto non-selective LB agar and incubated at 37°C overnight. Single colonies were collected, resuspended in 4 mL of 0.9% (wt/vol) saline solution, and grown on Oxoid Sensitest Agar. Plates were stamped using an antibiotic disc stamper and incubated at 37°C overnight. The following day, the annular radius of the inhibition zone surrounding each disc was measured. All resistance profiles were interpreted as resistant or susceptible according to the CLSI 2019 guidelines (31).

### Construction of rifampicin mutants in recipient cells

In conjugation assays, the recipient cells were made rifampicin resistant to allow the counterselection of transconjugants. Briefly, recipients were grown by spreading 100 µL of overnight culture on agar supplemented with rifampicin (50 µg/mL). Colonies that grew were purified by isolating single colonies on agar supplemented with rifampicin (100 µg/mL) to confirm their resistance profile and prevent contamination, as previously described (14).

### Plasmid DNA extraction

Plasmid DNA was extracted from 75 mL of overnight culture using the Promega PureYield Plasmid Midi-prep System (Cat. no. A2495) with minor modifications to the manufacturer’s protocol for purification by centrifugation. Briefly, changes included adding 600 µL of nuclease-free water to the column and heating it at 40°C for 1 h before the elution step. After extraction, plasmid DNA was quantified using a Thermo Fisher NanoDrop One.

### Electro-transformation assays

A single colony of the recipient strain was picked and grown in LB media overnight at 37°C. The following day, the culture was inoculated into fresh media in a 1:200 dilution and grown at 37°C with shaking at 250 rpm to reach the appropriate optical density (OD_600_) reading on a spectrophotometer instrument. The number of cells in each batch was estimated using an OD_600_ of 1.0, corresponding to 8 × 10^8^ cells/mL in *E. coli* cultures. Cells were pelleted at 5,000 × *g* and washed three times using ice-cold 10% (vol/vol) glycerol. Washed cells were resuspended in residual glycerol (approximately 100 µL) and stored on ice for electroporation.

Electro-transformations were conducted using conditions described by Chen et al. (32) (i.e., 0.2 cm cuvettes, 2.5 kV, 25 µF, and 200 Ω) in a BioRad MicroPulser Electroporator using the Ec2 setting (one pulse, 2.5kV). The appropriate volume of competent cells was added to a BioRad 0.2 cm electroporation cuvette and mixed with the appropriate amount of plasmid DNA. For all transformation experiments, we used the small plasmid pRAY* (6078 bp; carried by strain D36; GenBank no. CP012954.1). Following electroporation, the cells were immediately suspended in 3 mL of pre-warmed (37°C) LB media. Cells were grown at 37°C and 250 rpm for 2 h. The cells were serially diluted 1:10 and plated on LB agar supplemented with 30 µg/mL kanamycin. Following overnight incubation, colonies were counted and the electro-transformation efficiencies calculated as the average of three independent biological replicates (each with three technical replicates) using the following formula: number of colonies × dilution factor divided by amount of DNA electroporated in micrograms (µg).

To optimize electroporation conditions, culture volumes ranging from 5 to 300 mL were grown to an OD_600_ of 0.5–0.6 (4–5.1 × 10^8^ cells/mL) and concentrated to ~100 µL, yielding ~2.2 × 10^9^ to 1.5 × 10^11^ cells for transformation. Cultures were tested at both the exponential phase (OD_600_ 0.5–0.7) and the stationary phase (OD_600_ >1) following subculturing. Plasmid DNA was tested at either 25, 50, or 100 ng. Transformation was also performed with varying volumes of competent cells (50 or 80 µL), equivalent to approximately 2 × 10^7^ and 4 × 10^7^ cells, respectively. Normality of the data was tested using a Shapiro-Wilk test in GraphPad Prism v10.2.0. For data following a normal distribution, statistical significance was calculated using unpaired *t*-tests, while for data not following a normal distribution (i.e., stationary phase ATCC17978 cells), significance was calculated using a Mann-Whitney test. A *P*-value< 0.05 was considered significant in both tests.

### Natural transformation assays

Natural transformations were done using the low-salt method as previously described (33). Briefly, a single colony of the appropriate recipient strain was grown in 5 mL of LB media overnight at 37°C and 250 rpm. The following day, 1 µL of the culture and 500 ng or 100 ng of donor gDNA was added to 200 µL of LB without sodium chloride (10 g/L Tryptone and 5 g/L yeast) (33). The mixture was grown for 4 h at 37°C and 200 rpm. After incubation, 100 µL of the transformation mix was spread on LB agar without sodium chloride and supplemented with the MIC of kanamycin. Following overnight incubation, colonies were counted and the transformation values calculated as the number of colonies divided by amount of DNA added in micrograms (µg). Three natural transformation assays were conducted with three biological replicates (each with three technical replicates), and the average value was recorded.

### Conjugation (mating) assays

Conjugation assays were done on solid media using the classic mating assays described previously (9) with some modifications. Briefly, a single colony of the recipient strain and the donor strain (carrying the plasmid) was picked and grown in 5 mL of selective LB medium overnight at 37°C with shaking at 250 rpm. Subsequently, 100 µL of overnight culture from both the donor and recipient strains was mixed and incubated overnight on non-selective LB agar. Cells were then harvested in 1 mL of 0.9% (wt/vol) saline, serially diluted (10⁻²–10⁻⁹), and spot-plated on selective medium. Finally, 100 µL of the optimal dilutions (those yielding well-separated colonies) was spread on selective agar plates and incubated overnight at 37°C. Colony-forming units (CFU) on plates selective for donor and transconjugant strains were counted, and the efficiency of conjugation was calculated as “transconjugants per donor” using the formula: (CFU of transconjugants × dilution factor)/(CFU of donors × dilution factor). The reported values represent the mean from three independent biological replicates.

To optimize conjugative transfer conditions, we tested additional parameters, including the impact of the ratio of recipient to donor and temperature, using the AB307-0294^RifR^ (recipient) and ACICU (donor) combination, given that this pair showed the highest transfer values compared to other pairs tested in the previous round. To assess the impact of the modified conjugation conditions, three independent donor and recipient colonies were used, and each conjugation was conducted in triplicate. The final transfer efficiency was calculated as the average of the replicates.

### Whole genome DNA extraction

Whole genome DNA (gDNA) was extracted using the Qiagen DNeasy PowerSoil Pro Kit (catalog no. 47014) with minor modifications to the manufacturer’s protocol to increase yield. Briefly, 1.8 mL of overnight bacterial culture was pelleted for 10 min at 14,500 × *g* and resuspended in the kit-provided solution CD1 (lysis buffer). The solution was transferred to a PowerBead Pro Tube and vortexed horizontally at maximum speed for 5 min. To remove RNA contamination, the supernatant was then treated with 2 µL of RNase A (20 mg/mL) and incubated for 15 min at room temperature. After washing the column with the kit-provided solution C5, it was air-dried for 2 min. The genomic DNA was eluted in 40 µL of nuclease-free water and quality-checked using a Qubit 4 (Thermo Fisher Scientific) and a NanoDrop One (Thermo Fisher Scientific).

### Oxford Nanopore sequencing and whole genome methylation analysis

Extracted genomic DNA was sequenced at the Ramaciotti Center for Genomics (UNSW Sydney, Australia) with Oxford Nanopore technology (GridION GXB03456) using the Rapid Barcoding kit (SQK-RBK114-96 kit). This resulted in a complete genome with an average 50-fold coverage, N50 6.42 kb, and 99.9% base calling success. Basecalling and demultiplexing were done using *Dorado* v0.8.3 (Oxford Nanopore Technologies, ONT, Oxford, UK) with the following parameters: *sup model (--sup), no trimming of barcodes (--no-trim*), and model arguments *4mC*, *5mC*_*5hmC*, and *6mA* to call the N4-methylcytosine, C5-methylcytosine, 5-hydroxymethylcytosine, and N6-methyladenosine-modified bases, respectively. The generated .*bam* file was indexed using *Samtools* v1.17 (34) and aligned to reference FASTA files using *Minimap2* v2.28 (35). Methylated bases were identified using the *MicrobeMod* v1.0.4 (36) “call methylation” function. Call methylation was run using default parameters and a percent cutoff of 0.66 for its *Streme* v.5.5.8 dependency. Methylated sites were identified in *Methylartist* using log-likelihood ratios, which estimate the statistical probability that a site is truly methylated. Finally, methylation data were visualized and plotted using the *Methylartist* v1.3.1 program (37). The 5mC, 5hmC, and 6mA methylation patterns in plasmids were visualized using the *Methylartist “locus”* function using the motif “CCGG” for both pA297-3 and pACICU2 plasmids, based on motifs identified in *MicrobeMod*.

### Identifying host genomic features affecting plasmid uptake

The CRISPR-Cas type of the strains used was determined using the *CRISPRCasFinder* online server with default parameters as previously described (38).

To identify the presence of phage regions, the *PHAge Search Tool with Enhanced Sequence Translation* (*PHASTEST*) online server was used (39). Recipient strain genomes were uploaded to the server in FASTA file format and run using the “pre-computed results” setting.

Restriction-modification systems were typed using *MicrobeMod* annotate RM function with default parameters (36). Detected genes were further investigated using various programs, including *BLASTp* to identify proteins, *tBLASTn* to confirm their presence across all recipient strains, and the sequence data stored in the REBASE database (40).

## RESULTS AND DISCUSSION

### Optimizing electro-transformation using ATCC17978^SuS^ and AB307-0294

**Fig 1:**
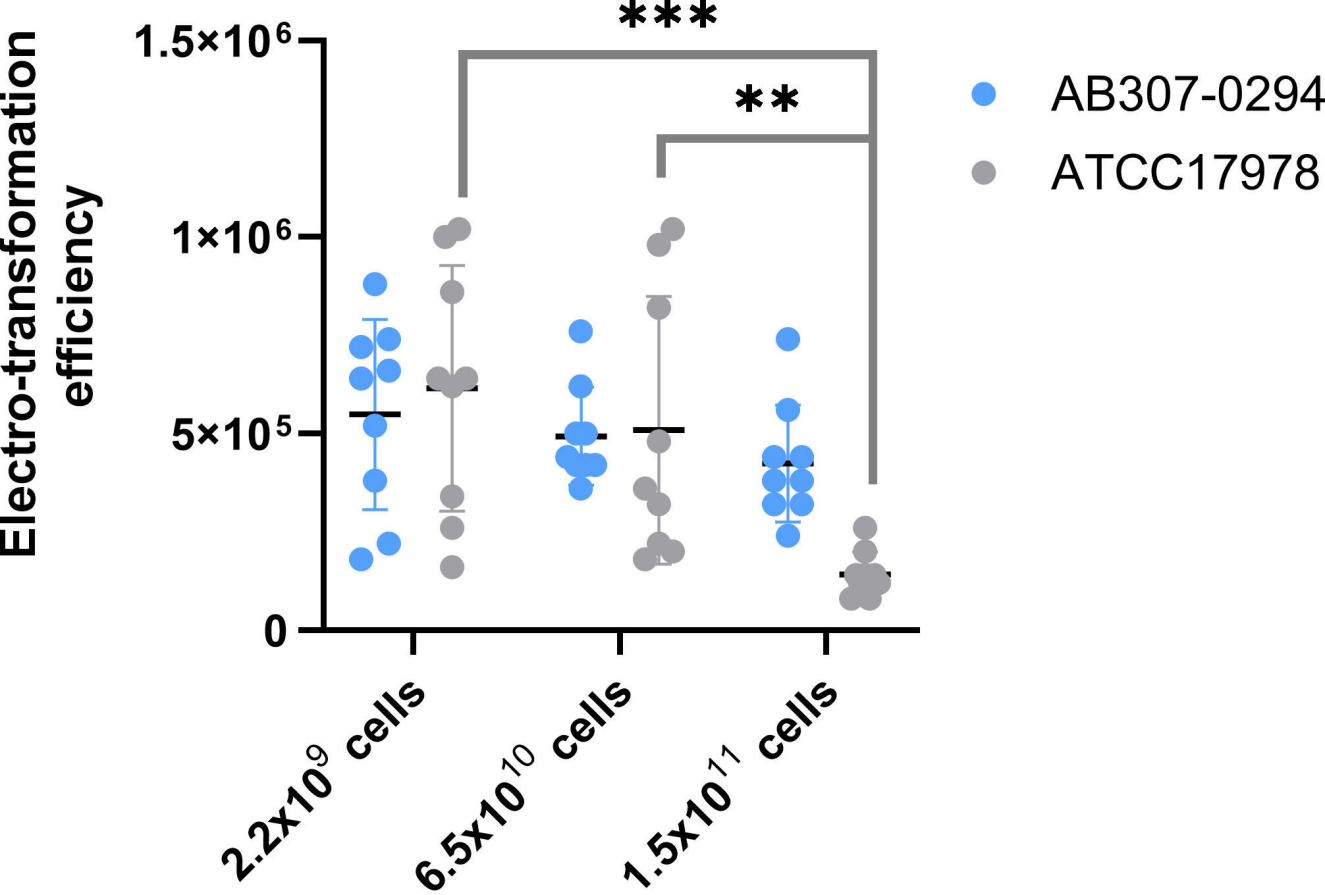
Electro-transformation efficiency of *A. baumannii* strains after changing the number of recipient cells made competent. Coloured dots represent the obtained electro-transformation efficiencies, and black** **bars represent the average value of each strain; the average was calculated using nine replicate tests. Error bars were calculated based on nine replicate tests, using the standard deviation.  Electro-transformation efficiency is measured on the y-axis in transformants/µg of DNA.* *

Two *A. baumannii* strains, a pAB3 cured and therefore sulfonamide susceptible version of the ATCC17978 strain (41) and AB307-0294 (21), were used as recipients to assess the effect of varying electroporation conditions, as these strains are commonly accessible in research laboratories. Using the plasmid pRAY* (carried by strain D36, GenBank no. CP012954.1), we systematically tested key parameters, including culture volume, growth phase, density of competent cells, and DNA amount. Competent cells were prepared from 5, 150, and 300 mL cultures, containing approximately 2.2 × 10⁹, 6.5 × 10¹⁰, and 1.5 × 10¹¹ cells, respectively. Across all conditions, plasmid uptake remained broadly similar for both strains, although ATCC17978^SuS^ showed significantly higher transformation efficiency with the smallest culture volume (5 mL) compared to the larger cultures (*P* = 0.0004). Consequently, subsequent optimization used this starting volume (~2.2 × 10⁹ cells) (Fig. 1).

**Fig 2:**
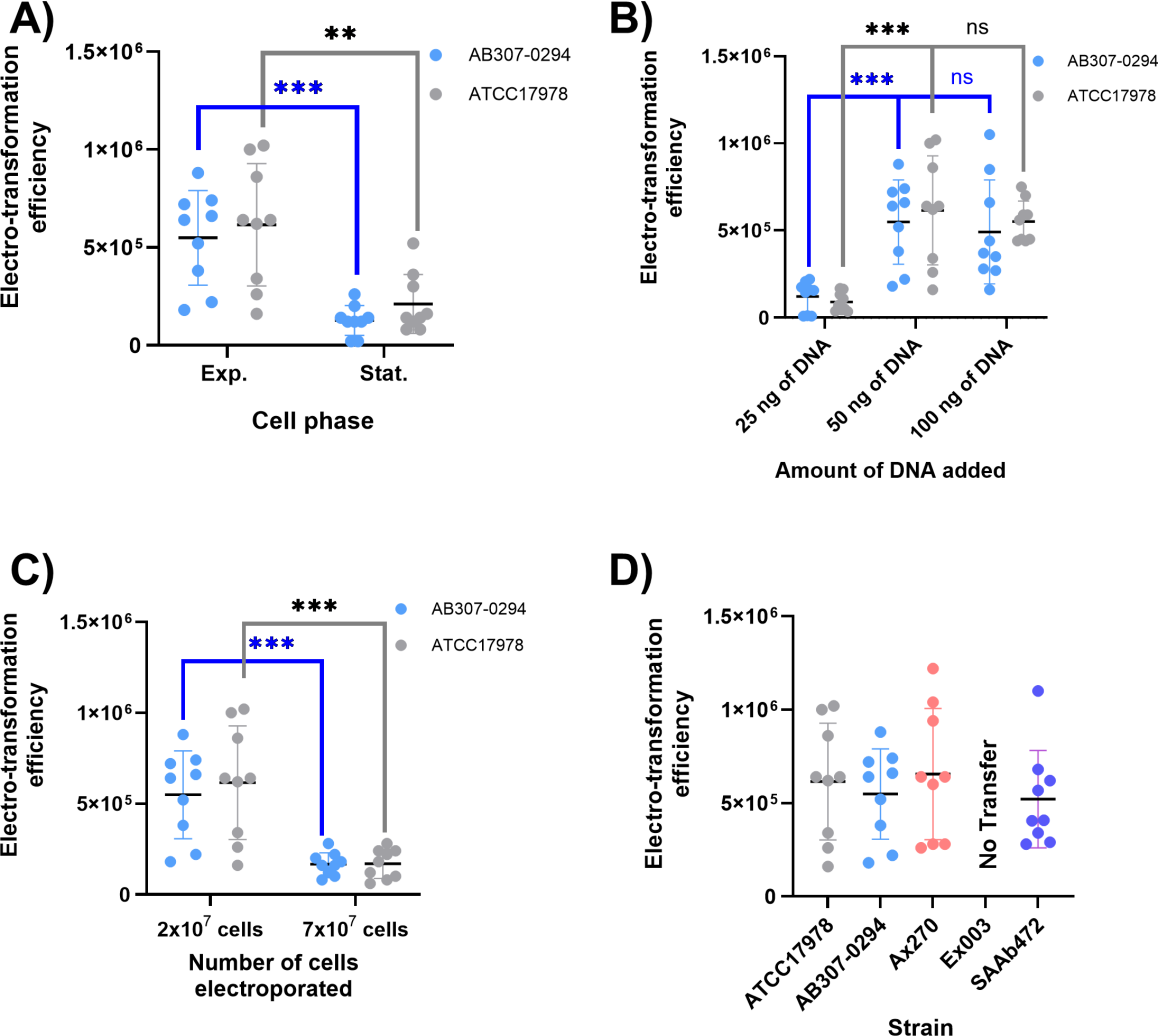
Electro-transformation efficiency of *A. baumannii* strains after changing experiment parameters. Scatter dot plots represent the obtained electro-transformation efficiencies, and black bars represent the average electro-transformation efficiency. The average was calculated based on nine replicate tests, and error bars were calculated using the standard deviation. Gray dots and error bars represent strain ATCC17978, blue dots and error bars represent strain AB307-0294, red dots and error bars represent strain Ax270, and purple dots and error bars represent strain SAAb472. Error bars were calculated based on nine replicate tests, using the standard deviation.  Parameters changed in each test are listed on the x-axis, and electro-transformation efficiency is measured on the y-axis in transformants/µg of DNA. (**A**) Electro-transformation efficiency by growth phase with 50 ng of DNA and 2 × 10^7^ competent cells. (**B**) Electro-transformation efficiency by plasmid DNA added using 2 × 10^7^ competent cells in the exponential phase. (**C**) Electro-transformation efficiency by competent cell density in the exponential phase with 50 ng of DNA. (**D**) Electro-transformation efficiency by strain using 2 × 10^7^ competent cells in exponential phase and 50 ng of DNA. * *

We next compared cells harvested at exponential (OD_600_ 0.5-0.7) versus stationary phase (OD_600_ >1). Both strains exhibited higher transformation efficiencies during exponential growth: AB307-0294, 5.49 × 10⁵ versus 1.27 × 10⁵ transformants/µg DNA (*P* = 0.0002) and ATCC17978^SuS^, 6.16 × 10⁵ versus 2.11 × 10⁵ (*P* = 0.0031) (Fig. 2A). These results indicate that exponential phase cells generally support more efficient plasmid uptake, although Yildirim et al. (18) observed a higher plasmid DNA uptake at a different OD_600_ (6.0; stationary phase) for pWH1266/ATCC17978, suggesting that this parameter may be different for each individual plasmid/strain combination.

Next, we tested the impact of plasmid DNA quantity (25, 50, and 100 ng) on electroporation efficiency. In both strains, 50 ng yielded an electroporation efficiency approximately half a log higher than that obtained with 25 ng of DNA (statistically significant; *P* = 0.0002–0.0003) (Fig. 2B). However, there was no statistically significant difference between 50 and 100 ng (*P* = 0.53–0.56), although the average efficiency appeared slightly higher with 50 ng. Yildirim et al. (18) found that 25 ng of pWH1266 can produce the highest efficiency (4.3 × 10^8^ transformants/μg DNA), which again suggests that the difference, compared to our findings, might be due to the individual plasmid/strain combination tested.

We also tested different numbers of competent cells used for electroporation using pellets with approximately 2.2 × 10^9^ cells. Experiments with volumes lower than 50 µL or higher than 80 µL produced low transfer values; hence, they were not further investigated. On average, 2 × 10^7^ (in a total volume of 50 µL) of competent cells resulted in more efficient electro-transformations (Fig. 2C) for both strains tested. In ATCC17978^SuS^, an efficiency of 6.16 × 10^5^ transformants/µg of DNA was obtained by using 50 µL of competent cells. In contrast, under the same conditions, the electro-transformation efficiency dropped significantly to 1.67 × 10^5^ transformants/µg of DNA when 80 µL of competent cells (7 × 10^7^ cells) was used (*P =* 0.0008). Overall, for both ATCC17978^SuS^ and AB307-0294, and under the conditions used here, pellets prepared from ~2.2 × 10⁹ cells (from 5 mL cultures) and regrown to exponential phase (OD₆₀₀ 0.5–0.7) yielded 2 × 10⁷ competent cells, which produced optimal results when transformed with 50 ng of pRAY.

### Strain-dependent variations in electro-transformation efficiency

After establishing the optimal conditions, we assessed pRAY uptake in a diverse set of *A. baumannii* recipients. These included SAAb472, recovered from wastewater in South Australia (26); Ex003, isolated from an artesian well water sample; and Ax270, obtained from a rectal swab of a cat (Table 1) (25). Notably, Ex003 carries an R3-type plasmid (encoding a Rep_3-type replication protein; pfam01051), while the other ST1 recipient (Ax270) did not contain any plasmid. Strain Ax270 exhibited a higher electro-transformation efficiency than other strains (7.33 × 10^5^ transformants/µg of DNA), while the electro-transformation rate was similar for both SAAb472 and AB307-0294 (~5.21 × 10^5^) (Table 3). Notably, Ex003 showed limited evidence of successful electro-transformation, indicating substantial strain-dependent variations in the electro-transformation efficiency of the small set we tested here (Fig. 2D). These varying results also suggest that there are genetic or epigenetic factors that could inhibit the successful uptake of plasmid DNA.

**TABLE 3:** Electro-transformation efficiencies in different *Acinetobacter* species^*a*^

Strains	Species	
AB307-0294	*A. baumannii*	5.49 × 10^5^
ATCC 17978^Sus^	*A. baumannii*	6.16 × 10^5^
Ax270	*A. baumannii*	7.33 × 10^5^
Ex003	*A. baumannii*	No transfer
SAAb472	*A. baumannii*	5.21 × 10^5^
SAAg309^*b*^	*A. gerneri*	4.4 × 10^4^
SAAt401^*b*^	*A. towneri*	3.20 × 10^4^
SAAj643^*b*^	*A. johnsonii*	2.67 × 10^1^
SAAc573^*b*^	*A. chinensis*	No transfer

^*a*^ using pellets prepared from ~2.2 × 10⁹ cells, regrown to exponential phase with 2 × 10⁷ competent cells and 50 ng of pRAY.

^*b*^ tested in host range assays.

Thus, for comparison, we also examined the ability of the recipient strains to take up plasmids via putative natural transformation events using the method previously described for *A. baumannii* (33). Among the five recipient strains tested, and under the conditions used here, only AB307-0294 and Ax270 successfully acquired pRAY*, indicating that the observed plasmid acquisition could be consistent with natural transformation.

Under conditions suitable for putative natural transformation, AB307-0294 yielded substantially fewer colonies compared with electroporation (~1 × 10² compared with 5.49 × 10⁵ transformants per μg of pRAY* DNA; Table S4). These results also indicate that differences in plasmid uptake efficiencies under conditions suitable for natural transformation likely reflect strain-specific biological factors or subtle differences in experimental conditions. However, determining the precise mechanism underlying plasmid uptake in our natural transformation assays (e.g., natural transformation vs. spontaneous plasmid uptake) will require targeted gene deletion and complementation experiments to establish whether these components (e.g., *comEC* and other genes) mediate the uptake events observed.

Overall, under the conditions tested here, lower starting culture volumes and competent cell concentrations enhanced electro-transformation efficiency, but the major determining factor (in both natural and electro-transformation) appears to be driven by the genetic background of the recipient strain, as it was also seen in natural transformation experiments.

### Strain-dependent variations in conjugative transfer

**Fig 3:**
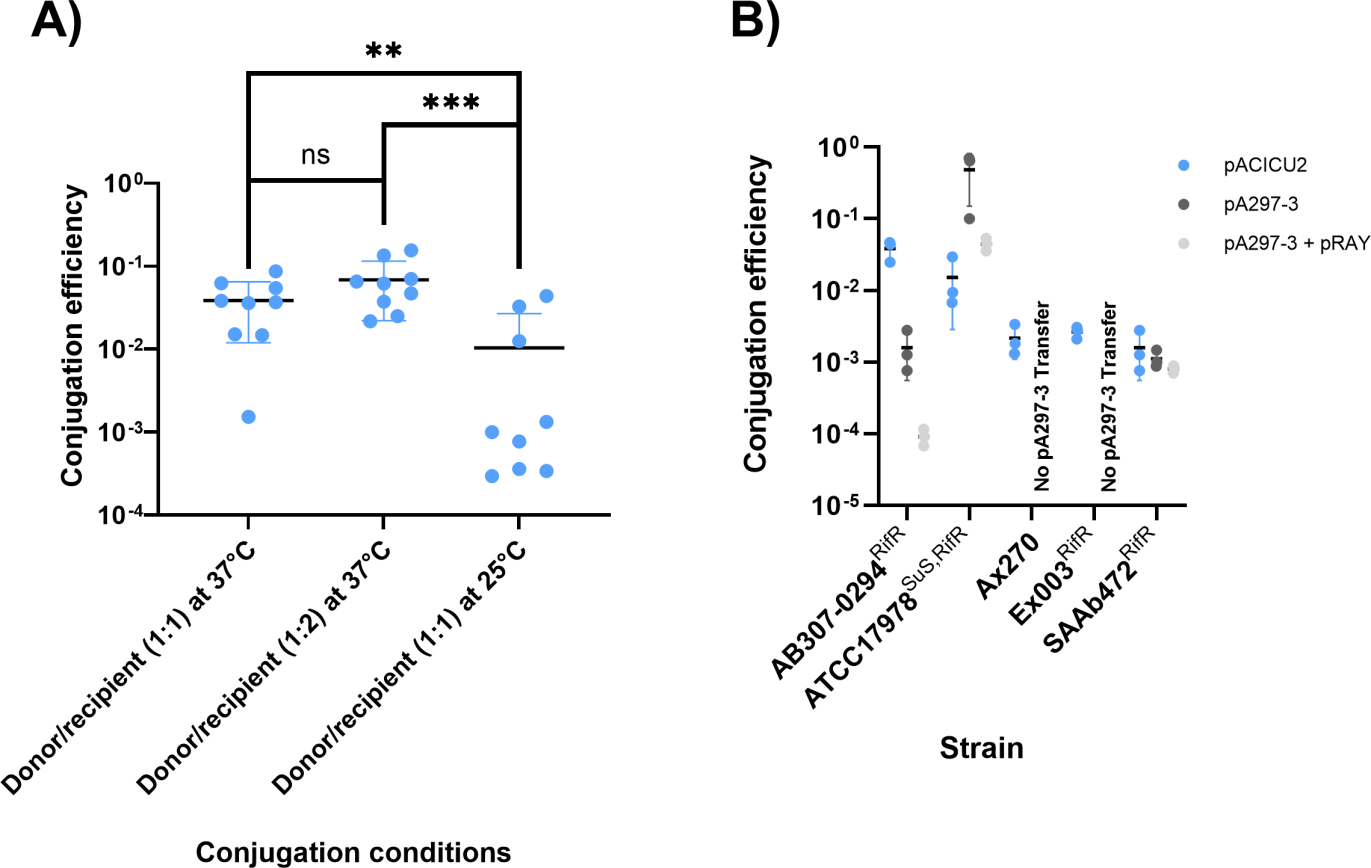
Conjugation efficiency of various plasmids in *A. baumannii *strains. (**A**) Optimization for the conjugative transfer of pACICU2 in strain AB307-0294. Colored blue dots represent the obtained conjugation efficiency of replicate tests, and black bars represent the average. The average was based on nine replicate tests. Error bars were calculated based on nine replicate tests, using the standard deviation. Efficiency is measured on the y-axis in transconjugants/donor, and test parameters are listed on the x-axis. (**B**) Conjugation efficiency of different plasmids into various *A. baumannii* strains. Colored dots represent the obtained conjugation efficiency of the plasmids, with blue dots representing pACICU2 transfer and gray dots representing pA297-3 transfer. Black bars represent the average, calculated using three replicate tests, and error bars are calculated using the standard deviation. Efficiency is measured on the y-axis in transconjugants/donor, and strains are listed on the x-axis.

To test the effect of the parameters used for conjugative assays, we used AB307-0294^RifR^ because it lacks any native plasmid, making it a more suitable recipient for conjugation assays. Two donor strains, ACICU and A297, were used for conjugation assays, carrying the conjugative plasmids pACICU2 (~70 kb) (11) and pA297-3 (~200 kb) (14), respectively. These plasmids represent well-characterized, clinically relevant conjugative plasmids commonly associated with antibiotic resistance in *A. baumannii* and were selected to assess the transferability of distinct plasmid backbones across diverse recipient strains. Using parameters we previously described (14), a conjugation frequency of 3.85 × 10^−2^ transconjugants/donor was achieved for pACICU2 (Fig. 3A). Although it was statistically insignificant (*P*=0.1359) (Fig. 3A), we found that when the volume of the recipient strain is doubled (i.e., 2:1 recipient-to-donor ratio), the transfer frequency increases to 6.87 × 10^−2^ transconjugants/donor, likely due to more recipients being available to uptake the plasmids. We also tested the ambient temperature and found that conjugation efficiency changed to 1.04 × 10^−2^ transconjugants per donor on solid agar, lower than that at 37°C (*P* 0.0056–0.0008 Table S4). Overall, high pACICU2 conjugative transfer efficiencies of 3.85 × 10^−2^ and 1.52 × 10^−2^ transconjugants/donor were obtained using AB307-0294^RifR^ and ATCC17978^SuS,RifR^, respectively (blue dots in Fig. 3B). However, pACICU2 transferred to the three non-human/environmental strains with a lower efficiency, ranging between 1.60 × 10^−3^ and 2.65 × 10^−3^ transconjugants/donor (Fig. 3B), likely due to genetic (and epigenetic) differences in recipient cells.

We also tested an additional plasmid, pA297-3 (a 200 kb conjugative plasmid that carries *sul2* (14)) as a representative of a common conjugative plasmid type (5), using the conditions tested for pACICU2. To test the transferability of pA297-3, we used *A. baumannii* strain A297 as a donor, which also contains the small plasmid pA297-1 (pRAY*). We previously showed that pRAY* can be mobilized with pA297-3 at a high frequency (42). The conjugative transfer of pA297-3 and the mobilization of pRAY are significant in the context of AMR spread. Thus, we also tracked the co-transfer of pRAY (light gray dots in Fig. 3B) during the pA297-3 conjugation assays. ATCC17978^SuS, RifR^ acquired both pA297-3 and pRAY* (co-transfer) at high efficiencies of 4.83 × 10^−1^ and 4.46 × 10^−2^ transconjugants/donor, respectively. However, compared to ATCC17978^SuS, RifR^, the pA297-3 transfer frequency was much lower (1.98 × 10^−4^ transconjugants per donor and 9.17 × 10^−5^ for pRAY mobilization) when AB307-0294 ^RifR^ was used as a recipient. We also obtained a similar frequency for SAAb472^RifR^ (i.e., 1.12 × 10^−3^ and 8.05 × 10^−4^ for pA297-3 transfer and pRAY co-transfer, respectively; Fig. 3B). However, pA297-3 could not move into the strains Ax270 and Ex003^RifR^ after several attempts, again indicating extensive discrepancies in conjugative frequencies in the strains tested here.

### *Acinetobacter* plasmids exhibit a genus-specific host range

**Fig 4:**
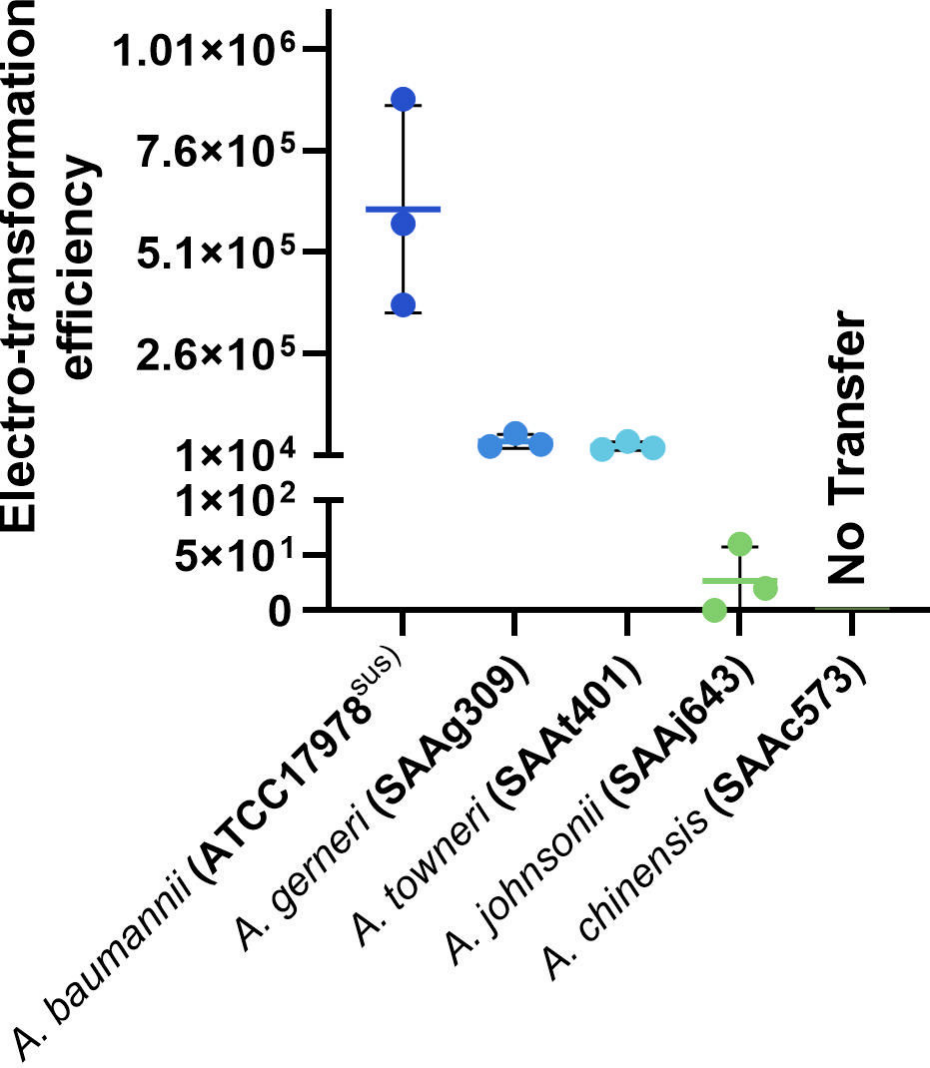
Electro-transformation efficiency of *Acinetobacter* strains at the exponential phase using 2 × 10^7^ competent cells and 50 ng of plasmid DNA (pRAY). Colored dots represent the obtained electro-transformation efficiencies, and black bars represent the average. The average was calculated based on three replicate tests and error bars using the standard deviation. Transformation efficiency is measured on the y-axis in transformants/µg of DNA and species, and strains are listed on the x-axis.

We recently showed that *A. baumannii* plasmids, including those tested here, have not been observed outside the *Acinetobacter* genus based on *in silico* analyses (6). Thus, using our optimized methods (see above), we tested the transferability of two clinically significant plasmids (pRAY* and pACICU2) into several non-*A. baumannii* strains, including *Acinetobacter chinensis* str. SAAc573, *Acinetobacter johnsonii* str. SAAj643, *Acinetobacter gerneri* str. SAAg309, and *Acinetobacter towneri* str. SAAt401 (Table 3), and four (*n* = 4) non-*Acinetobacter* recipients (including *E. coli* str. K-12*, P. aeruginosa* str. PAO1*, Stenotrophomonas maltophilia* str. CF13, and *Morganella morganii* str. 208.01^RifR^; Tables 2 and 3). All the non-*A. baumannii* strains but one (*A. chinensis* SAAc573) tested were able to uptake and maintain pRAY* stably using electro-transformation (Fig. 4). However, we observed a significant difference in transformation frequencies with *A. johnsonii* str. SAAj643 showed the lowest rate under the conditions tested (Table 3). We tested different growth conditions and noted that both *A. chinensis* SAAc573 and *A. johnsonii* SAAj643 grew better at temperatures between 25°C and 30°C. However, similar transfer results (compared to 37°C) were obtained, indicating an insignificant impact of temperature in transfer rates. The unsuccessful plasmid transfer into the *A. chinensis* strain is likely due to specific genetic factors that remain to be determined.

We also attempted the conjugative transfer of pACICU2 into the same non-*baumannii* species. However, only *A. gerneri* str. SAAg309^RifR^ was tested due to the availability of appropriate antibiotic selective markers. A low conjugative transfer rate in *A. gerneri* str. SAAg309^RifR^ was achieved (estimated 5.94 × 10^−4^ transconjugants/donor), indicating extensive variations compared to ATCC17978 and AB307-0294.

Several attempts to move pRAY* into *E. coli, P. aeruginosa, S. maltophilia,* and *M. morganii* strains (Table 2) were unsuccessful. Similarly, no conjugative transfer of pACICU2 was observed, indicating that none of these plasmids can replicate (or are stably maintained) in *E. coli, P. aeruginosa, S. maltophilia,* and *M. morganii* strains and that their host range is likely to be restricted within the *Acinetobacter* genus. However, more non-*Acinetobacter* strains and plasmids need to be tested to confirm this.

### Unique methylation profiles potentially influence transformation efficiency

**Fig 5:**
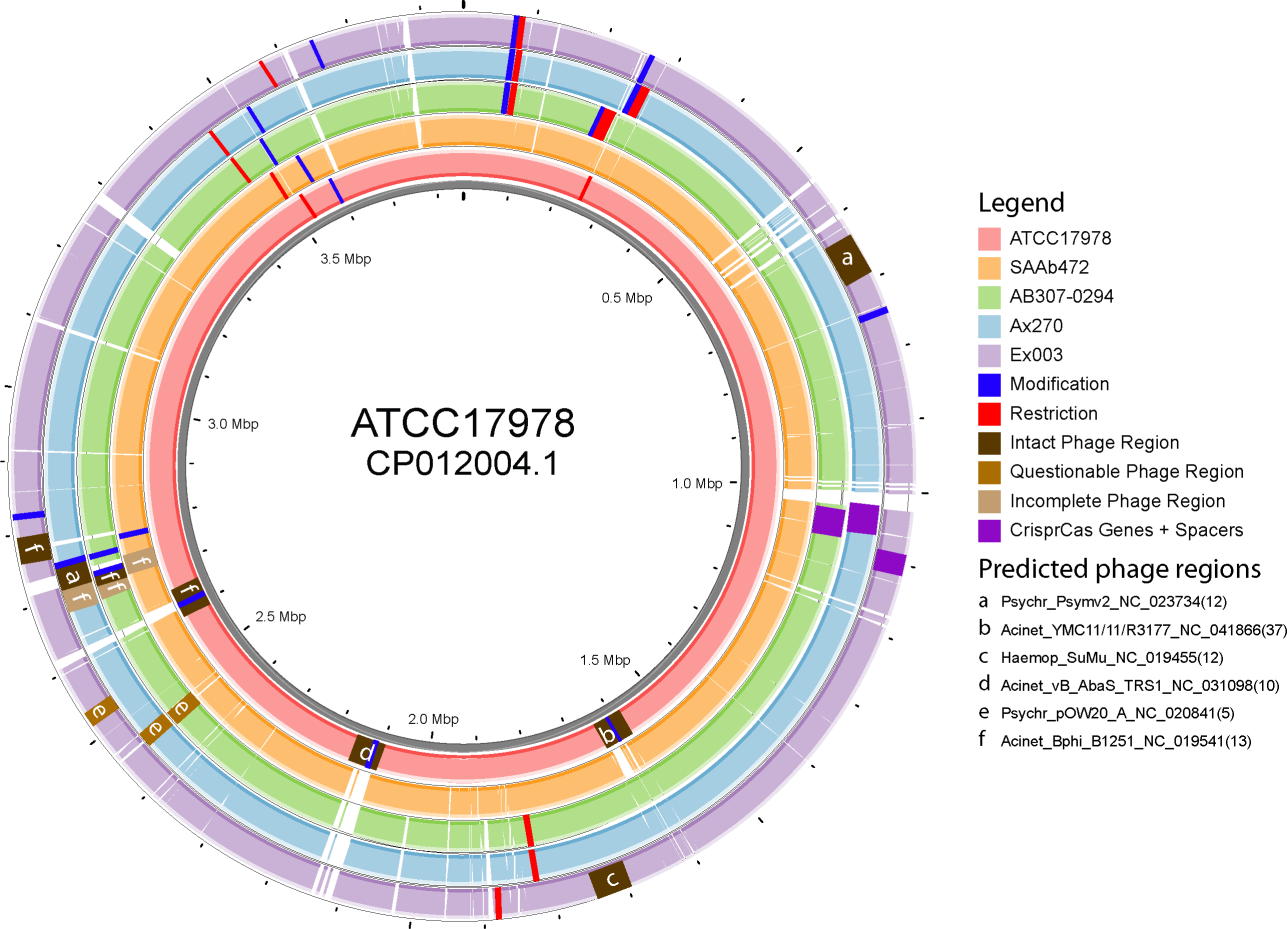
Comparative analysis of recipient *A. baumannii* strains used in this study. Colored rings represent BLAST searches comparing the recipient strains, with white spaces indicating gaps in the sequence alignment. Colored solid blocks on the rings represent features located in each of the strains and their approximate location. White letters seen inside the blocks represent phage regions discovered and their names, listed in the legend.

**Fig 6:**
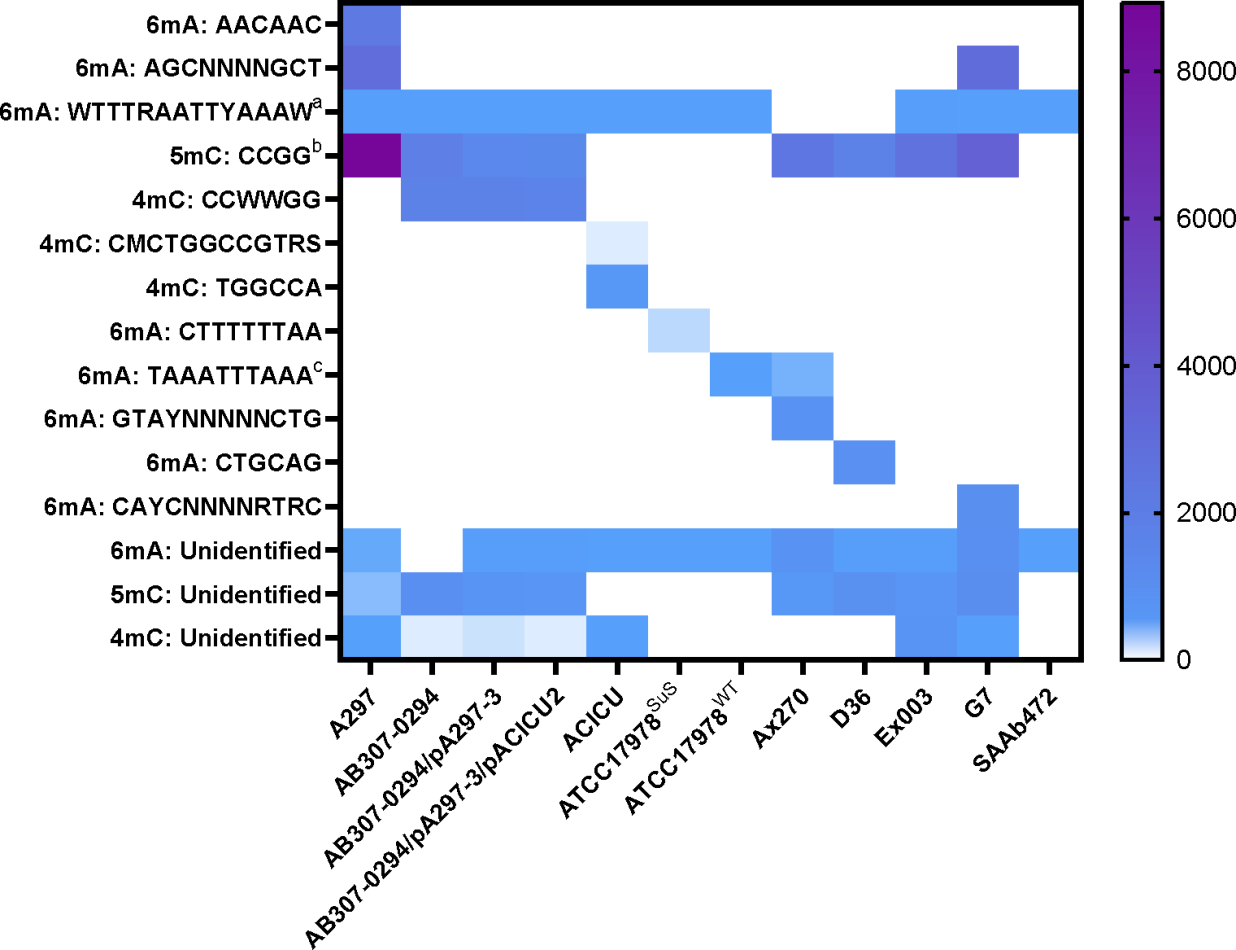
Distribution of methylation motifs within *A. baumannii *strains. Heatmap showing the abundance of different DNA modifications and motifs *in A. baumannii* strains. The type of DNA modification and motif is listed on the y-axis, with similar motifs combined. (a) Motif variants: TTTRAATTYAAA, WTTTNAATTNAAAW, and TTNAATTTAAAW. (b) Motif variants WNCCGGNW and WNCCGG. (c) Motif AAATTTAAATTT. Sites where methylation was detected and no motif was recognized have been listed as “Unidentified” on the y- axis. Strains and transconjugants are listed on the x-axis. Colored boxes represent the number of times a certain motif was detected.

DNA methylations have been shown to play a role in DNA acquisition rates (19). To further explore this, we used Oxford Nanopore technology (GridION) to sequence the wild-type *A. baumannii* strains and transconjugants generated in this study (Table 1). We searched for RM systems, analyzed methylated bases, and identified several RM systems in the study strains. There were three enzymes identified that were common to all recipient strains, including two methylases, 16S rRNA (guanine(966)-N(2))-methyltransferase RsmD and DNA adenine methylase, and one restriction enzyme, the molecular chaperone HtpG, suggesting that these are common enzymes in *A. baumannii*. However, we also found several RM systems that were present in only one of the study strains. For instance, in ATCC17978, there was both a unique restriction enzyme (restriction endonuclease PvuRts1I) and methylase (C-5 cytosine methyltransferase) identified (Fig. 5). Ax270 contained a unique complete operon, encoding a restriction enzyme (HsDR Family type I site-specific deoxyribonuclease), a methylase (Type I restriction-modification system), and an endonuclease helper with the specificity of methylation (restriction endonuclease subunit S). The presence of this operon, in only one strain (Ax270), might explain the failure to acquire pA297-3 by Ax270 (see above). Furthermore, Ex003 contained a unique Type-III site-specific DNA-methylase (Table S5). While it was not linked to a known restriction enzyme, there could be currently unrecognized or unknown restriction enzymes or mechanisms working with this methylase that could have contributed to the low levels of plasmid acquisition. Hence, we also used *MicrobeMod* (36) to identify DNA modifications (6mA, 5mC, 5hmC, and 4mC methylation) and the motifs recognized by methylases. All the strains sequenced, recipient and donor, had a unique methylation pattern, including the number and type of modification sites (Table 4), and motifs (Fig. 6). The total number of modified sites throughout the genomes ranged from 191 to 4,247 unique sites, indicating a wide variation in methylation patterns. In total, 18 unique methylation motifs were identified (Fig. 6). The majority of sites (*n* = 12) were for 6mA modifications, followed by three (*n* = 3) for 5mC and three (*n* = 3) for 4mC. Several motifs (*n* = 7) appeared in more than one strain, with the remainder being strain-specific.

**TABLE 4:** Number of methylation sites and RM systems found in *A. baumannii* strains

Strain	Chromosome/Plasmids	Size (bp)	6mA sites	5mC sites	4mC Sites	Complete RM operons	Single RM genes
A297	Chromosome	4,109,411	5,086	4,084	10	3	4
	pA297-1	6,078	7	5	0	0	0
	pA297-2	8,731	7	5	0	0	0
	pA297-3	200,633	194	529	0	1	0
AB307-0294	Chromosome	3,759,495	102	2,874	1,734	2	5
ACICU	Chromosome	3,919,274	100	0	519	0	8
	pACICU1b	24,268	0	0	0	0	1
	pACICU2	70,101	5	0	2	0	0
ATCC17978^SuS^	Chromosome	3,857,743	91	0	0	0	6
	pAB1	13,409	0	0	0	0	1
	pAB2	11,302	0	0	0	0	0
ATCC17978	Chromosome	3,857,743	109	0	0	0	6
	pAB1	13,409	8	0	0	0	1
	pAB2	11,302	0	0	0	0	0
	pAB3	148,955	0	0	0	0	0
Ax270	Chromosome	3,764,882	1,571	2,919	0	2	4
D36	Chromosome	4,063,596	995	2,663	0	2	7
	pD36-1	4,754	0	0	0	0	0
	pD36-2	6,078	0	2	0	0	0
	pD36-3	9,276	1	0	0	0	0
	pD36-4	47,457	12	1	0	0	1
Ex003	Chromosome	3,935,232	157	3,282	649	1	6
	pEx003	11,844	0	0	1	0	0
G7	Chromosome	4,072,048	4,840	4,654	25	2	6
	pAB-G7-1	8,731	5	0	0	0	0
	pAB-G7-2	70,100	51	27	0	0	0
SAAb472	Chromosome	3,809,697	91	0	0	0	3
AB307−0294/pA297-3	Chromosome	3,759,49	110	1,950	1,722	2	5
	pA297-3	200,633	8	50	5	1	0
AB307−0294/pA297-3/pACICU2	Chromosome	3,759,49	109	1,881	1,661	2	5
	pA297-3	200,633	8	43	2	1	0
	pACICU2	70,101	5	2	2	0	0

Furthermore, to examine if there was a link between plasmid transfer and methylation patterns, we analysed all five recipient strains. Ax270 showed the highest transformation efficiency of pRAY* with 7.33 × 10^5^ transformants/µg DNA. Analysis of the methylation patterns revealed that it had 4,490 modified sites, the majority of which (*n =* 2,919) were for 5mC modifications. Ex003, which failed to maintain the pRAY* plasmid, had a similar number of modifications (i.e., 4,089 modification sites in total, including 157 6mA, 3,282 5mC, and 650 4mC sites). The comparable quantity of sites indicates that specificity is more likely determined by the nature of the modifications rather than their number. This may have influenced the plasmid uptake efficiency of Ex003 or Ax270. Notably, Ex003 and Ax270, which failed to accept the plasmid pA297-3 via conjugation, each shared a unique motif combination (GTAYNNNNNCTG & TAAATTTAAA for 6mA modification & WNCCGG for 5mC in Ax270 and WNCCGG [5mC] & WTTTRAATTYAAAW [6mA] for Ex003; Fig. 6), suggesting that these specific combinations may have contributed to the failed conjugative transfer.

### Plasmid methylation profiles change as plasmids move into a new host

**Fig 7:**
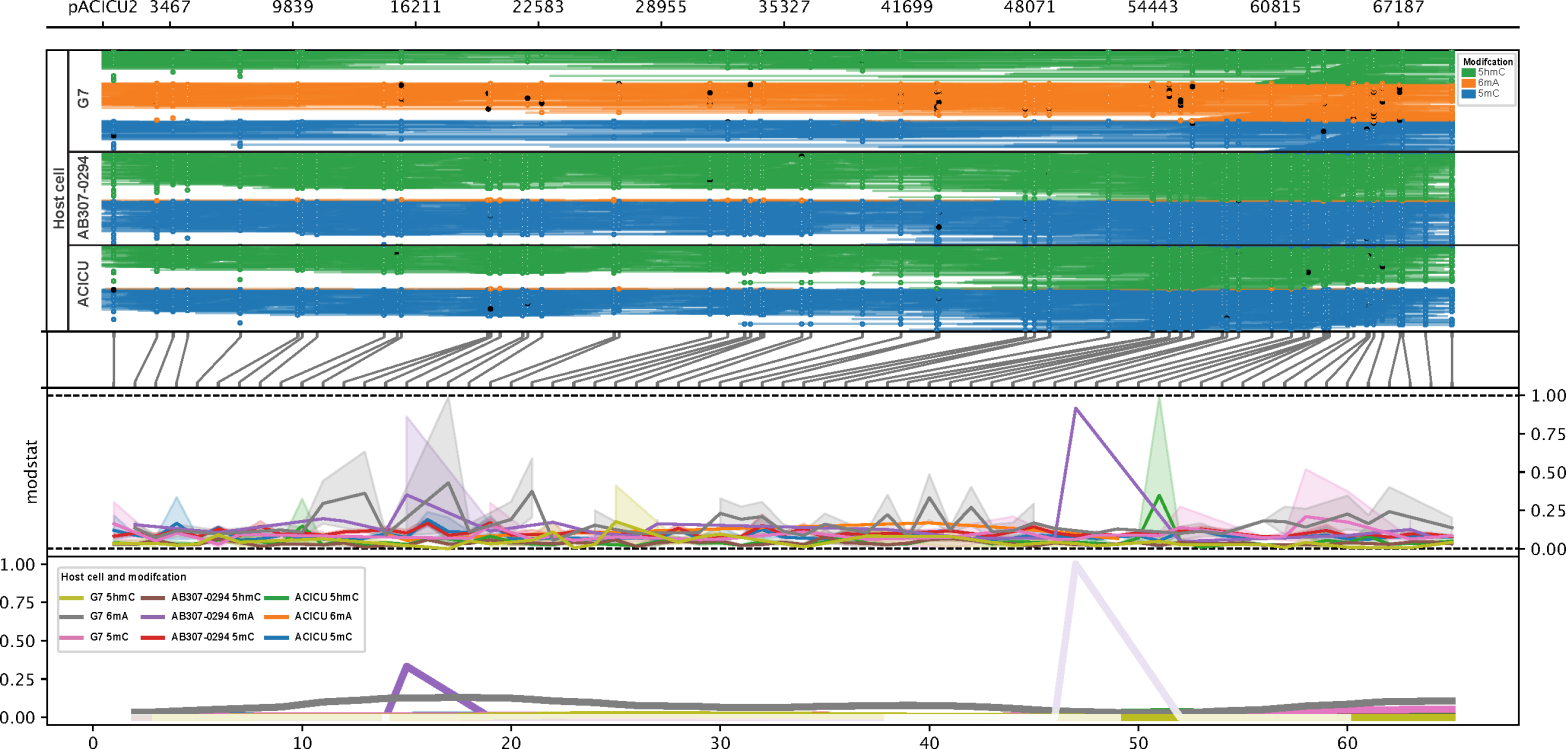
Comparison of methylation patterns in plasmid pACICU2. Methylartist output showing methylation patterns observed in pACICU2 when the host is changed. The top window shows individual reads, color-coded by the type of methylation observed (green = 5hmC, orange = 6mA, and blue = 5mC). Black dots on the reads indicate a site of methylation, while white dots show a site where methylation has not occurred. The second window matches the methylation sites in the reads to the corresponding log-likelihood ratio. The third window is the raw log-likelihood ratios (statistical tests calculating the statistical significance and probability that a site is genuinely methylated, with 1 being highly likely to be methylated). The bottom window shows the logs smoothed as a fraction plot.

To further investigate plasmid methylation profile variations, we analysed the methylation patterns in plasmids when grown in various hosts. Hence, we also sequenced a set of transconjugants, generated with AB307-0294 as the recipient cell, using Oxford Nanopore technology (GridION) (Table 4). First, we examined the conjugative plasmid, pACICU2 (70,101 bp), in three different hosts (Fig. 7). This plasmid is naturally found in strain ACICU, and when grown in its natural host, we identified seven modification sites (*n =* 2 4mC and *n =* 5 6mA, Table 4). Interestingly, when the plasmid was grown in a new host, AB307-0294, the modification sites were almost identical with *n =* 2 4mC and *n =* 5 6mA sites, and the addition of *n =* 2 5mC sites (Table 4). Interestingly, when comparing the types of modifications identified to those found in the host cells, there were no 5mC sites identified in the natural host, ACICU (Fig. 6), suggesting that the new host influences the sites of methylation seen in the plasmid. To further investigate the influence of the host, we studied an almost identical plasmid (with only five SNP differences compared to pACICU2), pAB-G7-2 (70,100 bp), whose natural host is G7 (Table 1). There were 78 modification sites identified on pAB-G7-2 in its natural host, indicating the host cell influences the pattern on the plasmid. To further support this, and consistent with pAB-G7-2’s unique methylation profile in its natural host (G7), we could not transfer pAB-G7-2 (using its natural host [strain G7] as a donor) to any recipient cells after several attempts, suggesting that the high methylation likely impairs acquisition by providing specificity to its original host. We observed that in the natural host (ACICU), pACICU2 had a lower quantity of 5mC methylated reads (black dots on blue reads, Fig. 7) and a lower quantity of 5mC peaks (blue peaks, Fig. 7). Supporting this, we did not identify any 5mC sites or motifs in the plasmid (Table 4). Contrasting this, an increase in both methylated reads and peaks was observed when the host was changed to AB307-0294, supporting the idea that the new hosts introduced 5mC modifications to the plasmid. Furthermore, the spread of the methylated sites in the plasmid shows the variation in the methylation profile when the host cell is changed (Fig. 7).

**Fig 8:**
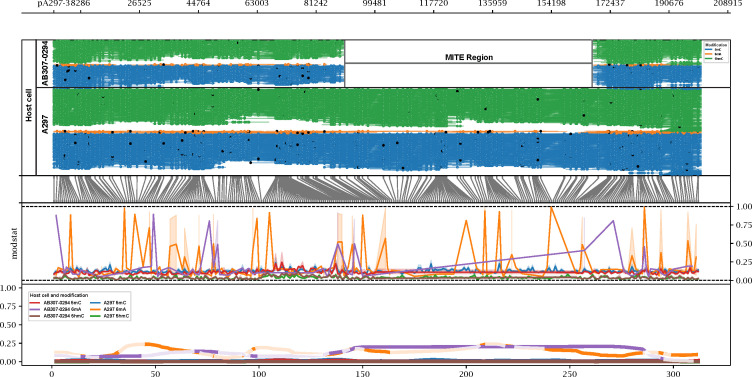
Comparison of methylation patterns in plasmid pA297-3. Methylartist output showing methylation patterns observed in pA297-3 when the host is changed. The top window shows individual reads, color-coded by the type of methylation observed (green = 5hmC, orange = 6mA, and blue = 5mC). Black dots on the reads indicate a site of methylation, while white dots show a site where methylation has not occurred. The second window matches the methylation sites in the reads to the corresponding log-likelihood ratio. The third window is the raw log-likelihood ratios (statistical tests calculating the statistical significance and probability that a site is genuinely methylated, with 1 being highly likely to be methylated. The bottom window shows the logs smoothed as a fraction plot.

Moreover, we observed similar changes in methylation profiles when the large conjugative plasmid, pA297-3, was in different hosts. When in its natural host (A297), it had 723 DNA modification sites, including *n =* 194 6mA and *n =* 529 5mC sites (Table 4). During conjugation, an 80 kb region was lost due to the recombination of identical miniature inverted-repeat transposable element copies (Fig. 8). The loss of this region could account for the lower number of identified methylation sites, with 244 sites (175 5mC and 69 6mA) located within this region in the wild type. However, there was a total loss of 660 sites across the plasmid when grown in host AB307-0294. In AB307-0294, we identified 63 modified sites including *n* = 8 6mA, *n* = 50 5mC, and *n* = 5 4mC sites in pA297-3. Notably, no 4mC sites were observed when it was grown in its original host, but *n* = 5 4mC sites appeared when grown in its new host, AB307-0294, consistent with the low number of identified 4mC sites (*n =* 10, Table 4) in the A297 chromosome and high number (*n =* 1,722) in the AB307-0294 chromosome (Table 4). pA297-3 also had *n* = 194 6mA sites in its natural host, while it only had *n* = 8 sites when it was in AB307-0294 (Fig. 8), indicating the host can influence the plasmid methylation pattern.

The altered methylation patterns indicate that the host exerts a significant influence on plasmid methylation, which in turn appears to be linked to the plasmid’s transfer efficiency. These findings tie into those by Vesel et al. (19), where identical plasmids were propagated in different hosts before extraction and transformation. They also found that the efficiency was higher if the plasmid was extracted from the original host and retransformed when compared to those extracted from a different host. They also performed an analysis of the methylome and found that different host strains have unique patterns (19), suggesting that each host will influence the methylome of any plasmids maintained within, thus uniquely modifying the plasmid. Together, our analysis of modification site distribution on the plasmid during host transition also demonstrates clear variations in peak locations and abundance. These findings indicate that plasmid methylation profiles are directly affected by the genetic differences of their host cells.

### Additional genetic differences in recipient cells

With the extensive differences in plasmid transfer frequencies, we further analyzed the recipient strains used in this study (Table 1) to identify additional genomic differences that may be linked to the plasmid uptake variations observed. Several major genetic differences were found (Fig. 5). The system of clustered regularly interspaced short palindromic repeats (CRISPR) and Cas proteins (CRISPR/Cas) has been recorded to act as an immune system for bacterial cells against incoming foreign DNA (43). These systems are often detected by identifying the palindromic repeats and the Cas proteins involved. Using *CRISPR-CasFinder* (38), we found that three of the recipient strains contained a CRISPR/Cas system (Table 5). All three of these strains are closely related, belonging to the ST1 group. Notably, analysis of the spacers found in two of these strains, Ax270 and Ex003, revealed that a 32 bp spacer “TTTGATAGCTAAAGTAGAAATCAAAGTCGCAA” was an exact match to a region in the plasmid pA297-3 (positions 40,059–40,090 in GenBank no. CP178357), indicating a previous invasion of the plasmid. Additionally, in conjugation assays, neither of these strains was able to obtain and stably maintain the plasmid. The lack of plasmid uptake suggests that both Ax270 and Ex003 have an active CRISPR-Cas system that can recognize and prevent the entry of pA297-3. Recent studies have characterized CRISPR-Cas diversity in *A. baumannii*, showing that certain subtypes are associated with reduced plasmid content and may limit horizontal gene transfer (44, 45). Our observation that Ax270 and Ex003 are resistant to pA297-3 uptake is consistent with these reports, supporting a functional role of CRISPR-Cas in restricting plasmid acquisition. Additionally, AB307-0294 also had a low plasmid uptake efficiency for pA297-3, suggesting that the low acquisition rate might be due to other factors, given that the spacer sequence was different from Ax270 and Ex003.

**TABLE 5:** CRISPR-Cas types detected in *A. baumannii* strains studied here^*c*^

Strain	CAS type	Spacers	Cas genes
ATCC17978	None found	–	*–*
ACICU	None found	–	*–*
A297	General-Class1^*a*^	53	*cas1326, csy123*
AB307-0294	IF^*b*^	45	*cas1326, csy123*
Ax270	IF	52	*cas1326, csy123*
Ex003	IF	54	*cas1326, csy123*
G7	None found	–	*–*
SAAb472	None Found	–	*–*

^*a*^ The repeat sequence unit is “TTTCTAAATGGCGTATGCCGCCATGAAC”.

^*b*^ The repeat sequence unit is “GTTCATGGCGGCATACGCCATTTAGAAA” in all strains with an IF-type Cas system.

^*c*^ "–" indicates not available.

Lysogenic bacteriophages will incorporate their DNA into a host’s genome. During this process, it is possible that the incorporated DNA could disrupt some cell functions (e.g., those involved in plasmid uptake). Hence, the presence of phage regions was identified using PHASTEST (32). In total, six different phage regions were identified, with all five recipient strains sharing regions for the Acinet_BPHI_B1251_NC_019541 phage (Fig. 5). Strain ATCC17978 had two unique regions, Acinet_YMC11/11/R3177_NC_041866 and Acinet_vB_AbaS_TRS1_NC_031098, being 50 kb and 35 kb, respectively. Strain Ex003 also had a unique 46 kb phage region, Haemop_SuMu_NC_019455. Notably, Ex003 had a very low transformation efficiency and was unable to take up one of the plasmids. The presence of a unique phage region provides a possible reason for the variation in plasmid transfer efficiencies. However, each strain also contained a prophage region in different chromosomal positions, suggesting that another point of difference is likely to influence plasmid uptake functions (Fig. 5).

### Conclusion

*A. baumannii* plasmids are important molecules in the spread of ARGs throughout the species. Here, we demonstrated that both electro-transformation and conjugation are effective laboratory techniques for moving plasmids between cells. In electro-transformation assays, the ideal conditions require lower volumes of competent cells and lower amounts of DNA. In conjugative transfer, the ideal conditions require an increasing ratio of donor and recipient cells, such that more recipients are available to uptake plasmids. Here, we showed that many genetic and epigenetic factors, including the RM systems, are likely to impact the uptake of plasmids in *A. baumannii*. The wide range of host factors and variability among the recipient strains provides a likely explanation for the variation in plasmid transfer. We also showed that different strains are likely to include different DNA modification patterns, despite their close relationship (i.e., genomic differences and different plasmid uptake efficiencies observed among ST1 recipients). Furthermore, plasmids will take on the methylation patterns of their host. Although these variations were identified in the present study, additional research is required to comprehensively characterize the genetic and epigenetic influences on plasmid DNA transfer in *Acinetobacter*.

## Data Availability

The long-read genome sequence data generated for the analysis of methylated bases have been deposited in the GenBank/EMBL/DDBJ database and are publicly available under GenBank BioProject accession number PRJNA1382140.

## SUPPLEMENTAL MATERIAL

Supplemental tables spectrum.03487-25-s0001.pdf Tables S1 to S5.

ASM does not own the copyrights to Supplemental Material that may be linked to, or accessed through, an article. The authors have granted ASM a non-exclusive, world-wide license to publish the Supplemental Material files. Please contact the corresponding author directly for reuse.
