# Supplementary material for: Variations in plasmid transfer in Acinetobacter baumannii: insights from epigenetics, strain properties, and experimental conditions
Source: Microbiol Spectr. 2026 Apr 27;14(6):e03487-25. doi: 10.1128/spectrum.03487-25 (PMC13227989; doi:10.1128/spectrum.03487-25)
Supplement: Supplemental tables — Tables S1 to S5. [file spectrum.03487-25-s0001.pdf]

Supplementary Table 1. Antibiotic resistance profiles of bacterial strains used in this project[#]

Species	Strain	IPM10*	AMP25	CTX30	MEM10	CAZ30	SAM20	TOB10	SPT25	GEN10	NET30	NEO30	KAN30
A. baumannii	ACICU	3	0	0	5	0	2	1	0	2	0	3	1
A. baumannii	A297	15	0	5	12	7	5	5	7	3	8	4	1
A. baumannii	AB307_0294	15	2	5	1	7	10	5	2	5	5	6	5
A. baumannii	ATCC17978[sus]	16	5	6	11	6	17	7	5	7	7	7	7
A. baumannii	Ax270	13	6	7	12	8	12	10	5	9	10	9	10
A. baumannii	Ex003	13	6	6	11	7	10	8	5	9	8	7	9
A. baumannii	SAAb472	20	5	7	8	6	15	8	3	10	12	8	8
A. baylyi	ADP1	15	5	8	10	3	11	7	6	8	10	9	8
A. towneri	SAAt401	15	5	8	7	4	10	4	3	6	8	6	3
A. gerneri	SAAg301	15	4	8	7	5	10	5	3	7	8	7	7
A. chinensis	SAAc573	13	5	11	7	6	15	7	6	10	10	8	9
A. johnsonii	SAAj643	17	6	10	9	3	13	7	6	8	10	8	9
P. aeruginosa	PAO1	10	0	3	6	12	0	7	3	6	6	4	1
S. maltophilia	CF13	0	0	3	0	0	0	0	0	10	5	7	2
M. morganii	208.01	7	5	15	7	12	5	7	5	8	10	8	7
E. coli	DH5α	16	16	18	15	15	11	12	9	12	15	11	13

Species	Strain	STR25	AMK30	SUL100	RIF30	TMP5	CR030	NAL30	CIP5	FFC30	CHL30	TET30
A. baumannii	ACICU	2	2	0	6	0	0	0	0	0	0	5
A. baumannii	A297	2	8	0	10	0	6	8	12	0	1	1
A. baumannii	AB307_0294	2	5	10	8	0	3	0	7	2	2	7
A. baumannii	ATCC17978[sus]	3	5	11	7	0	7	7	10	0	0	10
A. baumannii	Ax270	7	9	10	6	4	6	0	7	-[+]	0	7
A. baumannii	Ex003	6	8	10	6	4	5	7	9	-[+]	0	7
A. baumannii	SAAb472	3	8	15	10	0	6	10	15	2	1	14
A. baylyi	ADP1	9	8	12	10	0	3	9	10	7	-[+]	8
A. towneri	SAAt401	3	5	11	8	1	0	7	10	-[+]	-[+]	10
A. gerneri	SAAg309	3	5	12	7	1	0	7	12	-[+]	-[+]	10
A. chinensis	SAAc573	10	9	10	7	2	4	4	7	8	9	3
A. johnsonii	SAAj643	9	8	13	8	2	1	2	9	10	6	7
P. aeruginosa	PAO1	5	7	6	4	0	6	1	13	4	4	2
S. maltophilia	CF13	7	7	10	12	0	0	0	0	0	0	5
M. morganii	208.01	7	7	10	5	10	14	10	15	10	12	10
E. coli	DH5α^	11	13	19	18	19	17	4	17	-[+]	-[+]	-[+]

[#]Numbers indicate the inhibition zone radius in mm

+ Antibiotics were not available at time of testing

*Antibiotics and their concentration (µg) used in assay: Imipenem (IPM10), Ampicillin (AMP25), Cefotaxime (CTX30), Meropenem (MEM10), Ceftazidime (CAZ30), Ampicillin/Sulbactam (SAM20), Tobramycin (TOB10), Spectinomycin (SPT25), Gentamicin (GEN10), Netilmicin (NET30), Neomycin (NEO30), Kanamycin (KAN30), Streptomycin (STR25), Amikacin (AMK30), Sulphamethoxazole (SUL100), Rifampicin (RIF30), Trimethoprim (TMP5), Ceftriaxone (CRO30), Nalidixic Acid (NAL30), Ciprofloxacin (CIP5), Florfenicol (FFC30), Chloramphenicol (CHL30), Tetracycline (TET30)

Supplementary Tale 2. Transformation efficiencies using different conditions

Strain	Phase	Volume	Comp cell	DNA	Rep 1 Avg.	Rep 2 avg.	Rep 3 avg.	Avg.
Ab307-0294	Exponential	5	50	25	1.91E+05	1.64E+05	9.87E+03	1.22E+05
Ab307-0294	Exponential	5	50	50	6.33E+05	5.20E+05	4.93E+05	5.49E+05
Ab307-0294	Exponential	5	50	100	8.53E+05	2.37E+05	3.87E+05	4.92E+05
Ab307-0294	Exponential	5	80	50	2.47E+05	1.53E+05	1.07E+05	1.69E+05
Ab307-0294	Exponential	150	50	50	5.40E+05	4.93E+05	4.47E+05	4.93E+05
Ab307-0294	Exponential	300	50	50	4.40E+05	4.53E+05	3.80E+05	4.24E+05
Ab307-0294	Stationary	5	50	50	9.33E+04	9.33E+04	1.93E+05	1.27E+05
ATCC17978	Exponential	5	50	25	4.15E+04	7.55E+04	1.55E+05	9.07E+04
ATCC17978	Exponential	5	50	50	3.80E+05	5.80E+05	8.87E+05	6.16E+05
ATCC17978	Exponential	5	50	100	6.70E+05	4.93E+05	4.93E+05	5.52E+05
ATCC17978	Exponential	5	80	50	1.60E+05	1.87E+05	1.53E+05	1.67E+05
ATCC17978	Exponential	150	50	50	2.47E+05	8.27E+05	4.53E+05	5.09E+05
ATCC17978	Exponential	300	50	50	1.13E+05	1.13E+05	2.00E+05	1.42E+05
ATCC17978	Stationary	5	50	50	1.00E+05	3.40E+05	1.93E+05	2.11E+05
Ax270	Exponential	5	50	50	9.33E+05	2.73E+05	9.93E+05	7.33E+05
SAAb472	Exponential	5	50	50	3.68E+05	5.29E+05	6.67E+05	5.21E+05
Ex003	Exponential	5	50	50	No transfer			
SAAg309	Exponential	5	50	50	3.20E+04	3.60E+04	6.40E+04	4.40E+04
SAAt401	Exponential	5	50	50	4.40E+04	2.80E+04	2.40E+04	3.20E+04
SAAj643	Exponential	5	50	50	2.00E+01	6.00E+01	0	2.67E+01
SAAc573	Exponential	5	50	50	0	0	0	0

Supplementary Table 3. Conjugation frequencies under different conditions

Strain	Plasmid	Temperature	Donor/Recip	Conjugation	Conjugation	Conjugation	Average Conjugation Frequency
Ab307-0294	pACICU2	37°C	1:01	2.48E-02	4.67E-02	4.40E-02	3.85E-02
Ab307-0294	pACICU2	25°C	1:01	3.31E-04	1.03E-03	2.97E-02	1.04E-02
Ab307-0294	pACICU2	37°C	1:02	8.25E-02	5.65E-02	6.72E-02	6.87E-02
ATCC17978	pACICU2	37°C	1:01	6.79E-03	2.93E-02	9.41E-03	1.52E-02
Ax270	pACICU2	37°C	1:01	1.31E-03	1.82E-03	3.36E-03	2.16E-03
Ex003	pACICU2	37°C	1:01	2.79E-03	2.11E-03	3.06E-03	2.65E-03
SAAb472	pACICU2	37°C	1:01	2.77E-03	1.26E-03	7.63E-04	1.60E-03
SAAg309	pACICU2	37°C	1:01	1.04E-04	8.13E-04	8.68E-04	5.95E-04
Ab307-0294	pA297-3	37°C	1:01	8.00E-05	2.45E-04	2.68E-04	1.98E-04
ATCC17978	pA297-3	37°C	1:01	1.00E-01	7.00E-01	6.49E-01	4.83E-01
Ax270	pA297-3	37°C	1:01	No Transfer			
Ex003	pA297-3	37°C	1:01	No Transfer			
SAAb472	pA297-3	37°C	1:01	1.00E-03	1.48E-03	8.82E-04	1.12E-03
Ab307-0294	pA297-3 + pR	37°C	1:01	6.80E-05	9.09E-05	1.16E-04	9.17E-05
ATCC17978	pA297-3 + pR	37°C	1:01	5.33E-02	3.57E-02	4.48E-02	4.46E-02
Ax270	pA297-3 + pR	37°C	1:01	No Transfer			
Ex003	pA297-3 + pR	37°C	1:01	No Transfer			
SAAb472	pA297-3 + pR	37°C	1:01	8.89E-04	7.04E-04	8.24E-04	8.05E-04

Supplementary Table 4. Natural Transformation efficiencies

Strain	DNA added (ng)	Transformation Efficiency 1	Transformation Efficiency 2	Transformation Efficiency 3	Average Transformation Efficiency
Ab307-0294	500	56	54	25	45
Ab307-0294	100	102	120	186	136
Ax270	500	16	52	29	48
Ax270	100	100	213	300	204

Supplementry Table 5. Restirction/Modification genes found in Recipient strains

Strain	Operon	System type	Type of enzyn	Gene
Ab307-0294	RM Operon #	Type II	Restriction	ATP-binding protein
Ab307-0294	RM Operon #	Type II	Methylation	DNA cytosine methyltransferase
Ab307-0294	RM Operon #	Type IV	Restriction	5-methylcytosine-specific restriction enzyme B†
Ab307-0294	RM Operon #	Type IV	Restriction	McrC family protein
Ab307-0294	RM Operon #	Type II	Methylation	DNA-methyltransferase
Ab307-0294	Singleton #1	Type III	Restriction	DEAD/DEAH box helicase
Ab307-0294	Singleton #2	Type II	Methylation	DNA-methyltransferase
Ab307-0294	Singleton #3	Type II	Methylation	16S rRNA (guanine(966)-N(2))-methyltransferase RsmD
Ab307-0294	Singleton #4	Type II	Restriction	molecular chaperone HtpG
Ab307-0294	Singleton #5	Type II	Methylation	DNA adenine methylase
ATCC17978	Singleton #1	Type II	Restriction	restriction endonuclease PvuRts1I
ATCC17978	Singleton #2	Type II	Methylation	DNA cytosine methyltransferase
ATCC17978	Singleton #3	Type II	Methylation	methyltransferase domain-containing protein
ATCC17978	Singleton #4	Type II	Methylation	DNA cytosine methyltransferase
ATCC17978	Singleton #5	Type II	Restriction	molecular chaperone HtpG
ATCC17978	Singleton #6	Type II	Methylation	DNA adenine methylase
Ax270	RM Operon #	Type II	Restriction	ATP-binding protein
Ax270	RM Operon #	Type II	Methylation	DNA cytosine methyltransferase
Ax270	RM Operon #	Type I	Restriction	HsdR family type I site-specific deoxyribonuclease
Ax270	RM Operon #	Type I	Methylation	type I restriction-modification system subunit M
Ax270	RM Operon #	Type I	Specificity	estriction endonuclease subunit S
Ax270	Singleton #1	Type III	Restriction	DEAD/DEAH box helicase
Ax270	Singleton #2	Type II	Methylation	16S rRNA (guanine(966)-N(2))-methyltransferase RsmD
Ax270	Singleton #3	Type II	Restriction	molecular chaperone HtpG
Ax270	Singleton #4	Type II	Methylation	DNA adenine methylase
Ex003	RM Operon #	Type II	Restriction	ATP-binding protein
Ex003	RM Operon #	Type II	Methylation	DNA cytosine methyltransferase
Ex003	Singleton #1	Type III	Methylation	DNA methyltransferase
Ex003	Singleton #2	Type II	Methylation	methyltransferase domain-containing protein
Ex003	Singleton #3	Type III	Restriction	DEAD/DEAH box helicase
Ex003	Singleton #4	Type II	Methylation	16S rRNA (guanine(966)-N(2))-methyltransferase RsmD
Ex003	Singleton #5	Type II	Restriction	molecular chaperone HtpG
Ex003	Singleton #6	Type II	Methylation	DNA adenine methylase
SAAb472	Singleton #1	Type II	Methylation	16S rRNA (guanine(966)-N(2))-methyltransferase RsmD
SAAb472	Singleton #2	Type II	Restriction	molecular chaperone HtpG
SAAb472	Singleton #3	Type II	Methylation	DNA adenine methylase
